# Nutrients and Metabolites as Signalling Molecules in Osteoclasts

**DOI:** 10.1007/s11914-026-00955-4

**Published:** 2026-02-09

**Authors:** Kavishadhi Chandrasekaran, Sitao Hu, Kara Farstad-O’Halloran, Killugudi Swaminatha Iyer, Haibo Jiang, Nathan Pavlos, Kai Chen

**Affiliations:** 1 School of Biomedical Sciences, University of Western Australia, Perth, WA 6009 Australia; 2 School of Molecular Sciences, University of Western Australia, Perth, WA 6009 Australia; 3 Department of Chemistry, University of Hong Kong, Hong Kong, China

**Keywords:** Osteoclast, Metabolites, Nutrient sensor, Immunometabolites, Epigenetics, Bone

## Abstract

**Purpose of Review:**

This review aims to highlight the emerging concept that nutrients and metabolites act not merely as energy sources or biosynthetic precursors, but also as instructive signalling molecules in osteoclasts. While much is known about transcriptional and genetic pathways governing osteoclast differentiation and function, comparatively little attention has been given to the role of cellular metabolism and nutrient-sensing mechanisms. This review seeks to categorise key metabolites based on their signalling roles and examine how they influence osteoclastogenesis through metabolic, epigenetic, and inflammatory pathways.

**Recent Findings:**

Recent studies have demonstrated that nutrients such as glucose, amino acids, and lipids, along with their intermediary metabolites such as succinate, itaconate, α-ketoglutarate (αKG), S-adenosylmethionine (SAM), and acetyl-CoA, regulate osteoclast formation and function by modulating signalling cascades and epigenetic landscapes. These molecules engage nutrient sensors (e.g., aldolase, mTORC1, CPT1) and transcriptional regulators (e.g., NFATc1, PPARs), while also affecting chromatin structure, inflammatory responses, and organelle dynamics.

**Summary:**

Osteoclast metabolism is tightly linked to cellular fate through nutrient-sensing and metabolite-driven signalling. Elucidating these pathways will reshape our understanding of osteoclast regulation and help identify new metabolic targets for treating bone diseases.

## Introduction

Osteoclasts are specialised, multinucleated cells responsible for bone resorption and play a critical role in skeletal development and homeostasis [1, 2]. The osteoclast life cycle—including differentiation, resorption, apoptosis, and fission—is tightly regulated by a network of cytokines, transcription factors, and cell–cell interactions within the bone marrow niche. While significant advances have been made in elucidating the genetic and molecular determinants of osteoclast biology, emerging studies have shed light on an underexplored regulatory layer: cellular metabolism. Osteoclasts are metabolically active cells that undergo extensive metabolic reprogramming in order to meet the high energy demands associated with differentiation and function [3, 4].

Historically viewed as a passive process supplying energy and biosynthetic precursors, metabolism is now recognised as a dynamic and active modulator of cell fate and function [5–7]. In this context, nutrients (e.g., glucose, amino acids, and lipids) and their metabolites not only act as energy substrates but also serve as important messenger molecules that transduce signalling and influence cell behaviours [5, 8, 9]. This conceptual shift reframes our understanding of metabolism from a background process to a central regulatory hub, with nutrients and metabolites as signalling molecules in both intra- and intercellular contexts.

These signalling metabolites exert their effects through various mechanisms, including post-translational modifications (PTMs), engagement with metabolite-sensing proteins, allosteric modulation of enzymes, and regulation of protein–protein interactions [5]. The ability of metabolites to act as modulators and real-time cues makes them uniquely suited to regulate dynamic biological processes such as osteoclast differentiation and bone resorption. Compared to well-established research fields such as cancer, liver biology, and neuroscience, the utilisation and importance of individual nutrients and metabolites as signalling molecules in bone resident cells such as osteoclasts remain in their infancy. Osteoclast maturation involves complex biological processes—including cell-to-cell fusion, cytoskeletal and membrane reorganisation, and acidification of the resorption lacuna—all that demand substantial ATP production, redox homeostasis, and biosynthetic precursor availability.

In this review, we highlight emerging insights into how nutrients and metabolites act as instructive signals that shape osteoclast differentiation and function. We categorise these signalling nutrients and metabolites based on their predominant or emerging roles into three groups: (1) Sensor-responsive nutrients/metabolites, which are detected by specialised sensors to reflect nutrient availability and subsequent trigger metabolic and functional adaptations during osteoclastogenesis; (2) Immunometabolites, which shape inflammatory and immune-like responses in osteoclast precursors; (3) Epigenetic metabolites, which modulate chromatin architecture and gene transcription. While we focus on representative mechanisms within each category, these examples only begin to capture the breadth and complexity of metabolite-mediated regulation in osteoclast biology.

## Sensor-Responsive Nutrients: More than Fuelling Osteoclasts

**Fig. 1:**
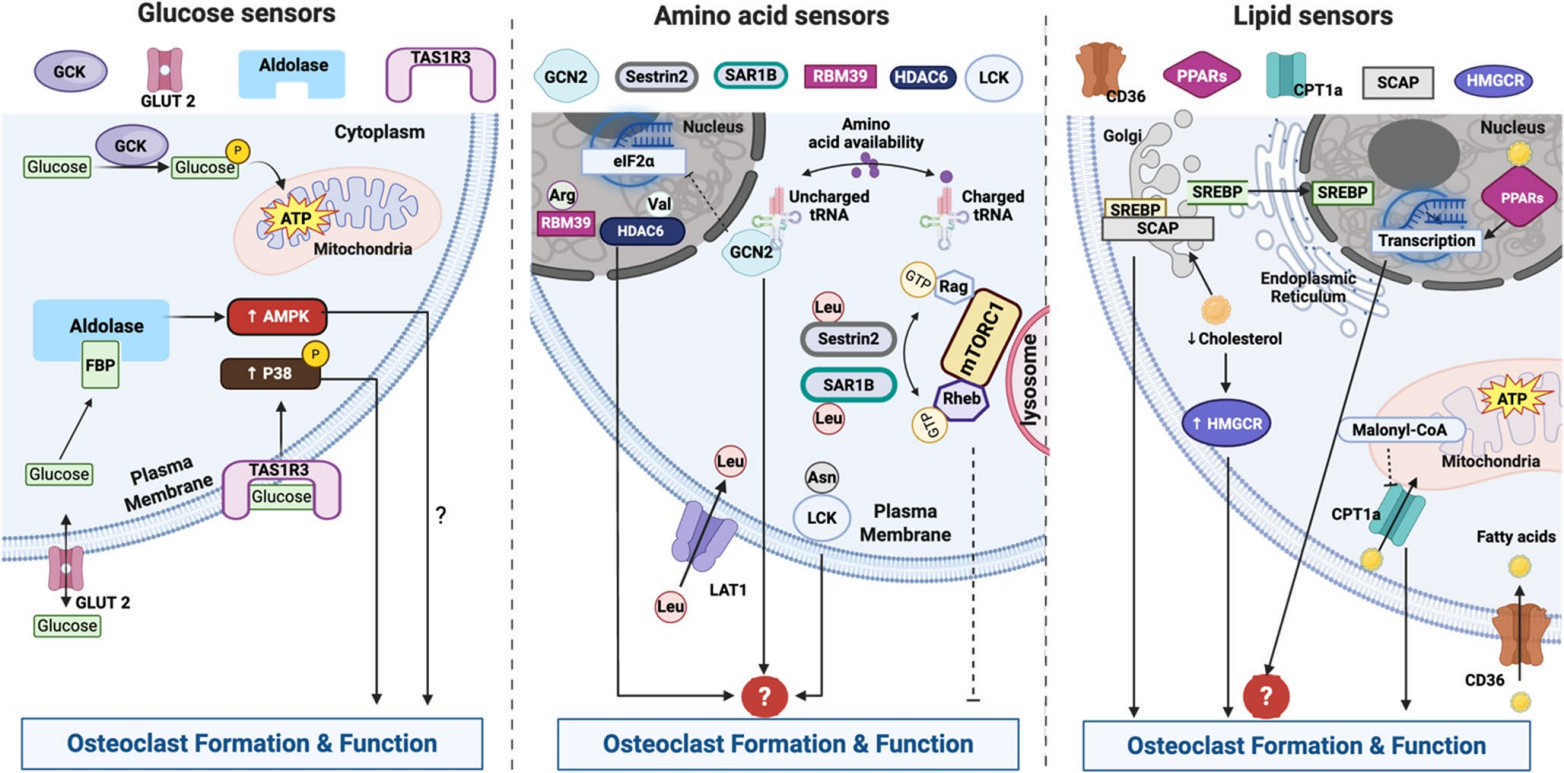
Nutrients and their sensors regulate osteoclast formation and function. Arg, Arginine; Asn, Asparagine; CPT1, Carnitine Palmitoyltransferase 1; eIF2α, Eukaryotic Initiation Factor 2 Alpha; FBP, Fructose-1,6-Bisphosphate; GCK, Glucokinase; GCN2, General Control Nonderepressible 2; GLUT2, Glucose Transporter 2; HMGCR, 3-Hydroxy-3-Methylglutaryl-CoA Reductase; LAT1, L-type Amino Acid Transporter 1; Leu, Leucine; PPARs, Peroxisome Proliferator-Activated Receptors; SCAP, SREBP Cleavage-Activating Protein; TAS1R3, Taste Receptor Type 1 Member 3; Val, valine. Created in BioRender. https://BioRender.com/rp8o1m3

Glucose, amino acids, lipids, and their derivatives are not merely energy substrates or structural components; their intracellular and extracellular fluctuations are sensed by specialised proteins known as nutrient sensors [8–10]. From this perspective, nutrient availability and metabolite levels serve as instructive cues sensed by these proteins to activate downstream signalling pathways (Table 1). Despite growing interest, this remains an underexplored area in osteoclast biology. Here, we summarise the current understanding of nutrient sensors and their roles in osteoclast regulation (Fig. 1).

**Table 1:** Nutrients and sensors in osteoclasts

Nutrients	Sensors	Function and its relevance to osteoclasts
Glucose	GLUT2	A glucose transporter responds to high-glucose level and facilitates glucose import and export from cells [8, 11].
		Minimal expression in osteoclasts [12]
	GCK	A hexokinase becomes active under high-glucose conditions and is responsible for glucose phosphorylation and ATP production [13, 14].
		Upregulated during hypoxia-driven osteoclastogenesis [15].
	Aldolase	Fructose-1,6-bisphosphate (FBP) sensing modulates AMPK via V-ATPase complex assembly in response to both high and low glucose levels [16–18].
		Aldolase A is linked to low BMD and may promote bone resorption by interacting with V-ATPases at the ruffled border [19, 20].
	TAS1R3	A sweet taste receptor in the oral and extra oral tissues [21].
		Senses glucose levels and promotes osteoclast differentiation via p38 signalling; loss impairs function and reduces bone mass [22–24].
Amino acids	GCN2	Amino acid sensing via uncharged tRNAs and inhibits mRNA translation through eIF2α [25–28].
		Expression and functional role remain unclear in osteoclasts.
	mTORC1	Amino acid sensing through Rag and Rheb GTPases, which regulate its lysosomal activation state via nucleotide changes [29].
		Modulating NF-κB/NFATc1 signalling; mTORC1 overactivation impairs osteoclast function, while inhibition by rapamycin restores it [30, 31].
Lipids	CD36	A transmembrane sensor for extracellular fatty acid uptake [32].
		Critical for RANKL signalling and osteoclast formation [33–35].
	PPARs	Fatty acids activated transcription factors [36, 37].
		Show both inhibitory and stimulatory effects on osteoclastogenesis depending on context [38–42].
	CPT1	Transporting FAs into mitochondria for oxidation [43, 44].
		CPT1a links lipid metabolism to osteoclast function and bone loss [45–47].
	SCAP	Senses cholesterol levels to control SREBP activation; low cholesterol triggers SREBP cleavage and lipid synthesis [48].
		SCAP inhibition can suppress osteoclast differentiation and bone loss [49, 50].
	HMGCR	Senses cholesterol intermediates and is degraded via the ubiquitin-proteasome pathway to limit cholesterol synthesis [51].
		Statins can inhibit HMGCR activity and have been shown to suppress osteoclast formation [52, 53].

1. *Glucose*
  Glucose is utilised by osteoclast precursor cells to fuel oxidative phosphorylation and aerobic glycolysis during RANKL-mediated differentiation and consumed by mature osteoclasts for glycolysis during bone resorption [12, 54]. Genetic or pharmacologic inhibition of glucose uptake markedly suppresses osteoclastogenesis in vitro but produces only minor or sexually dimorphic effects on osteoclast number and bone mass in vivo [12]. These differences likely reflect metabolic plasticity, including the ability of osteoclasts to draw on alternative substrates and adapt to systemic and microenvironmental cues. Here, we focus on how glucose availability may be “sensed” and coupled to osteoclast signalling programs. Cells detect glucose availability through glucose sensors, including glucose transporter 2 (GLUT2), glucokinase (GCK), and aldolase. GLUT2 is a bidirectional, low-affinity glucose transporter that is most active when extracellular glucose concentrations are high, in contrast to high-affinity transporters such as GLUT1 and GLUT4 that remain active under fasting glycemic conditions [8, 11]. Notably, in osteoclast lineage cells, GLUT1 is considered the predominant glucose transporter supporting glucose entry, whereas GLUT2 appears to be minimally expressed [12]. This suggests that glucose sensing in osteoclasts is more likely mediated by intracellular, flux-coupled nodes rather than a canonical GLUT2-centered extracellular sensing module.
  Intracellularly, GCK is a hexokinase which becomes active only under high-glucose conditions and phosphorylates glucose to make glucose-6-phosphate (G6P) and initiate ATP production [13, 14]. GCK is shown to be upregulated during hypoxia-driven osteoclastogenesis in vitro [15]. Aldolase is a well-known glycolytic enzymes and recently has also emerged as an important glucose sensor that can respond to both high and low glucose levels by directly binding to the glucose-derived fructose-1,6-bisphosphate (FBP) [16–18]. Under high-glucose conditions, aldolase binds excess FBP, preventing the formation of the lysosomal V-ATPase–regulator complex and thereby suppresses AMP-activated protein kinase (AMPK) activation. Conversely, in low-glucose conditions, unbound aldolase facilitates the assembly of the V-ATPase complex and promotes AMPK signalling. Interestingly, *ALDOA* (encoding aldolase A) was identified as a genetic factor associated with bone mineral density (BMD) [19] and proteomic analysis of peripheral blood monocytes (PBMs) also revealed upregulation of aldolases in individuals with lower BMD [20]. Furthermore, aldolase can bind to vacuolar H⁺-ATPases (V-ATPases) and form a complex near the ruffled border that can contribute to bone resorption [20]. These studies imply an important role for aldolase in osteoclasts; however, precisely how glucose levels are sensed and how downstream signalling is modulated by aldolase during osteoclastogenesis remains unclear. More recently, taste receptor type 1 member 3 (TAS1R3), traditionally known as a sweet taste receptor in the oral and extra oral tissues (e.g., intestine, brain) [21], has been found to be highly expressed in osteoclasts and to contribute to osteoclast differentiation by sensing glucose levels and modulating p38 phosphorylation [22]. Loss of TAS1R3 expression impairs osteoclast function and leads to high bone mass [23, 24]. Together, these glucose-sensing mechanisms may play a critical role in osteoclast formation and function, representing potential therapeutic targets for osteoclast-related bone disorders.
2. *Amino Acids*
  Amino acids are indispensable building blocks for proteins, and the deficiency of one cannot be simply compensated by another. Therefore, cells must efficiently detect and respond to the fluctuations in amino acid levels [8, 25]. Osteoclast differentiation is accompanied by substantial changes in amino acid metabolism [55]. GCN2 (general control nonderepressible 2) and mTORC1 (mechanistic target of rapamycin complex 1) are two critical pathways that are highly responsive to the amino acid abundance. GCN2 is activated by amino acid deficiency (uncharged tRNAs) when requisite nutrients are limiting, whereas mTORC1 primarily reports amino acid sufficiency/abundance to modulate anabolic signalling. During protein synthesis, amino acids are loaded to their cognate tRNAs, and uncharged tRNAs can be detected by GCN2, triggering downstream inhibition of mRNA translation via eIF2α (eukaryotic translation initiator factor 2 α) [25–27]. GCN2 senses amino acid deficiency and regulates the DNA damage response, linking amino acid availability to cell fate decisions [28]. GCN2 ablation causes an osteoblast and skeletal phenotype, while serum C-terminal telopeptide of type I collagen (CTX) remains unchanged [56], suggesting that GCN2 is unlikely to be a major determinant of systemic osteoclast-mediated bone resorption under basal conditions.
  mTORC1 responds to amino acid sufficiency via Rag and Rheb GTPases, which control its recruitment to the lysosome. Amino acid-induced nucleotide changes in Rag determine whether mTORC1 is active or inactive [29]. More studies suggest that mTORC1 is a negative regulator of osteoclastogenesis by modulating the NF-κB/NFATc1 signalling. Activation of mTORC1, achieved by deleting TSC1—a negative regulator of mTORC1 that inhibits Rheb GTPase activity—in osteoclast precursors leads to severe defects in osteoclast maturation and bone resorptive function [30, 31]. In contrast, inhibition of mTORC1 using rapamycin can restore osteoclast differentiation and function, highlighting the importance of finely tuned mTORC1 activity in osteoclasts [31]. mTORC1 is not equally responsive to all amino acids; among them, leucine plays a particularly critical role in its activation [8, 57]. Leucine uptake via the L-type amino acid transporter 1 (LAT1) is essential for intracellular mTORC1 signalling, and LAT1 deficiency enhances osteoclast activation by impairing mTORC1 activity [58]. The direct cytosolic sensors for leucine, such as Sestrin2 [59, 60] and SAR1B [61], have been identified. Among them, Sestrin2 has been previously implicated to affect osteoclast differentiation although its effects remain controversial [62, 63]. In addition to leucine, mTORC1 is also responsive to other amino acids such as arginine, methionine, threonine, and glutamine; however, how their respective sensors influence osteoclast function via mTORC1 remains unclear. Beyond the mTORC1 and GCN2 pathways, several mTORC1-independent amino acid sensors have been recently identified—such as TAS1R1/3 (a glutamate sensor) [64], HDAC6 (a valine sensor) [65], RBM39 (an arginine sensor) [66], LCK (an asparagine sensor) [67]. For example, TAS1R1/3 have been linked to glutamate detection [64], and osteoclast metabolomics studies report a prominent increase in glutamate metabolism during osteoclast differentiation [55]. Moreover, glutamate can contribute to the TCA intermediate α-ketoglutarate (αKG), which supports epigenetic regulation during osteoclastogenesis [68] (discussed below). These findings raise intriguing questions about whether and how individual amino acids act as signalling molecules through their cognate sensors to modulate osteoclast differentiation and activity.
3. *Lipids*
  Lipids, including fatty acids (FAs) and sterols, are hydrophobic molecules essential for membrane structure, energy storage, and cellular signalling. Osteoclastogenesis is an energy-intensive process characterized by dynamic lipid reorganization during membrane fusion, for which lipid-sensing mechanisms are anticipated to play crucial roles [69, 70]. Here, we briefly review the metabolic sensors for FAs (i.e., CD36, PPARs, and CPT1) and cholesterol (i.e., SCAP and HMGCR) and their reported roles in osteoclasts. CD36, a transmembrane receptor well-known for its sensor role, governs extracellular FA uptake and utilisation [32], which is critical to support osteoclast formation [33–35]. Internally, FAs and their metabolites act through transcriptional FA sensors PPARs (peroxisome proliferator-activated receptors), including PPARα, PPAR β/δ, and PPARγ [36, 37]. While PPARs have been implicated in the regulation of osteoclastogenesis, their exact role(s) remain unclear with some studies report that activation of PPARs by FAs or specific agonists suppresses osteoclast formation [38, 39], whereas others suggest a pro-osteoclastogenic effect [40–42]. Carnitine palmitoyltransferase 1 (CPT1) is a rate-limiting enzyme in FA oxidation, catalysing the transport of FAs into mitochondria. CPT1 activity is inhibited by malonyl-CoA, a key intermediate in FA synthesis [43, 44]. CPT1a, a key CPT1 isoform, is a well-established mediator linking FA oxidation to osteoclast differentiation and function. Its deficiency impairs osteoclast activity and is associated with rheumatoid arthritis and bone loss [45–47]. However, in vivo Cpt1a loss produces a modest, female-restricted skeletal phenotype [45], similar to that reported in Cpt2-disrupted mice [71], suggesting context-dependent compensation and arguing against CPT1A as a dominant lipid “sensor” in physiological osteoclastogenesis. The specific compensatory FA sensors or pathways in osteoclast lineage cells in males remain to be defined. Cholesterols essentially constitute cellular membranes [72], for which the cholesterol-rich lipid rafts are crucial for transducing RANKL signalling during osteoclast formation and function [73, 74]. Cholesterol biosynthesis is tightly regulated by intracellular cholesterol levels which are sensed by two critical proteins, SREBP cleavage-activating protein (SCAP) [48] and HMG-CoA reductase (HMGCR) [51]. Within the endoplasmic reticulum (ER), SCAP binds to SREBPs, the transcription factors for cholesterol production. When cholesterol levels are high, SCAP–SREBP complex retains in the ER. In contrast, low cholesterol allows the complex to move to the Golgi, where SREBP is cleaved and its active domain translocates to the nucleus to enhance lipid synthesis. The SCAP inhibitor, Fatostatin, has been shown to inhibit osteoclast differentiation and bone loss [49, 50]. HMGCR, the rate-limiting enzyme in cholesterol synthesis, contains a sterol-sensing domain and undergoes rapid degradation via the ubiquitin-proteasome pathway when cholesterol intermediates accumulate [51]. Statins are HMGCR inhibitors and have been shown to suppress osteoclast formation [52, 53]. Together, these findings highlight that lipid-sensing mechanisms, through both FA and cholesterol sensors, are integral to osteoclast formation and function, offering potential therapeutic targets for osteoclast-related bone diseases.

## Immunometabolites: Linking Inflammation To Osteoclastogenesis

**Fig. 2:**
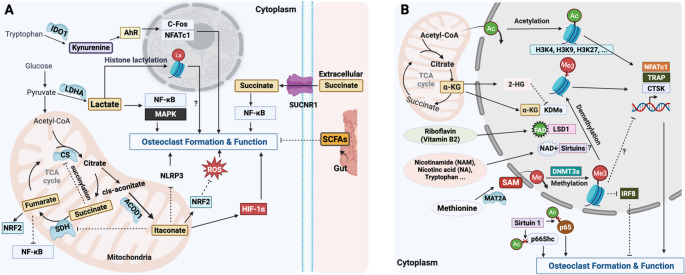
Metabolites in osteoclasts. (**A**) Immunometabolites and (**B**) epigenetic metabolites regulate osteoclast formation and function. 2-HG, 2-Hydroxyglutarate; AhR, Aryl Hydrocarbon Receptor; αKG, Alpha-Ketoglutarate; CS, Citrate Synthase; CTSK, Cathepsin K; Dnmt3a, DNA Methyltransferase 3 A; FAD, Flavin Adenine Dinucleotide; HIF-1α, Hypoxia-Inducible Factor 1 Alpha; KDM6B, Lysine Demethylase 6B; LSD1, Lysine-Specific Demethylase 1; NAD⁺, Nicotinamide Adenine Dinucleotide; NF-κB, Nuclear Factor Kappa-B; NRF2, Nuclear Factor Erythroid 2–Related Factor 2; ROS, Reactive Oxygen Species; SAM, S-Adenosylmethionine; SCFAs, Short-Chain Fatty Acids; SDH, Succinate Dehydrogenase; SUCNR1, Succinate Receptor 1 (GPR91); TCA, Tricarboxylic Acid Cycle. Created in BioRender. https://BioRender.com/eeccraq

Immunometabolites refers to metabolic intermediates that accumulate during immune cell activation and exert regulatory effects on immune responses [75–77]. While originating from studies in immune cells such as macrophages and T cells [78–81], these metabolites—such as succinate and itaconate—are increasingly recognised as key modulators in non-traditional immune players such as osteoclasts and their precursors, which share key developmental and functional pathways with myeloid lineages (Table 2; Fig. 2A).

**Table 2:** Metabolites as signalling regulating osteoclast formation and function

Metabolites	Major Sources	Mechanisms
Succinate	TCA cycle	Binding to SUCNR1 and enhances osteoclastogenesis via NF-κB activation [82, 83]; Succinylation of citrate synthase (CS) and reduces its enzymatic activity during osteoclastogenesis [84].
Itaconate	TCA cycle	Suppressing osteoclastogenesis by inhibiting SDH [85], repressing NLRP3 inflammasome [86], and activating NRF2 signalling [87].
Lactate	Glycolysis	Promoting osteoclastogenesis through activation of the NF-κB and MAPK pathways [88] and histone/protein lactylation [89].
Fumarate	TCA cycle	Downregulating NFATc1 and disrupting mitochondrial oxidative metabolism [90, 91].
Kynurenine	Tryptophan catabolism	Promoting osteoclast differentiation by activating AhR pathway and inducing downstream c-Fos/NFATc1 [92–95].
SCFAs	Gut microbiota	Inhibiting osteoclast differentiation via “gut-bone” axis [96–98]
αKG	TCA cycle	Enhancing osteoclastogenesis by facilitating NFATc1 transcription via H3K27me3 demethylation [68, 99]
		Regulating H3K9 demethylation at *Slc7a11* promoter, which enhances NRF2 binding and suppresses ROS level and inhibits osteoclast differentiation [100]
SAM	Methionine metabolism	A universal methyl donor methylation reactions [101, 102]; Mediating DNA methylation through Dnmt3a, and repressing anti-osteoclastic transcription factors [103].
Acetyl-CoA	Catabolic and anabolic metabolism	Increases histone acetylation and upregulates osteoclastogenic markers [104]; Mitochondrial acetyl-CoA production mediates CTSK expression and collagen degradation [105].
NAD⁺	Kynurenine pathway/ Salvage pathway	A cofactor for sirtuins which can deacetylate non-histone proteins such as p66Shc [106] and the p65 subunit of NF-κB [107], modulating osteoclast differentiation and function.
FAD	Riboflavin (Vitamin B2) metabolism	A cofactor for the lysine-specific demethylase 1 (LSD1) which demethylates histone and modulates osteoclastogenesis [108–111]
2-HG	Derivative of αKG	Inhibits αKG-dependent dioxygenases and induces epigenetic alteration [112, 113]

1. *Succinate*
  Succinate is a mitochondrial metabolite of the tricarboxylic acid (TCA) cycle, primarily generated through the conversion of α-ketoglutarate (α-KG) to succinyl-CoA, playing a central role in ATP production. Beyond its classical role in energy metabolism, succinate has emerged as an immunometabolite that bridges cellular metabolic state with immune activation. It exerts signalling functions by stabilising the transcription factor hypoxia-inducible factor-1α (HIF-1α) [114], binding to its cognate receptor SUCNR1 (also known as GPR91) [115, 116], and inducing post-translational protein modification by lysine succinylation. Dysregulated succinate signalling has been linked to inflammation, angiogenesis, and metabolic reprogramming in diseases such as cancer, obesity, and ischemia [117]. Recent evidence implicates succinate in promoting osteoclast activation and bone resorption, thereby contributing to pathological bone loss [82–84]. Guo et al. first demonstrated that under hyperglycaemic conditions, bone marrow stromal cells (BMSCs) release succinate, which binds to SUCNR1 on osteoclast lineage cells and enhances osteoclastogenesis via NF-κB activation, ultimately promoting bone loss [82]. Consistently, succinate derived from hypoxic dental pulp stem cells (DPSCs) was shown to stimulate osteoclast differentiation and induce root resorption, while genetic or pharmacological inhibition of SUCNR1 reduced osteoclast maturation [82, 83]. More recently, Yu et al. demonstrated that RANKL-induced disruption of the TCA cycle leads to intracellular succinate accumulation in osteoclast precursors. This, in turn, promotes succinylation of citrate synthase (CS) and reduces its enzymatic activity, contributing to the regulation of osteoclastogenesis [84]. Together, these findings position succinate as a multifaceted regulator of osteoclastogenesis, acting through both extracellular receptor-mediated signalling and intracellular metabolic reprogramming.
2. *Itaconate*
  Itaconate is a metabolite substantially upregulated in various immune settings [118, 119] as well as osteoclastogenesis. It is synthesised from the TCA cycle intermediate cis-aconitate by the enzyme aconitate decarboxylase 1 (ACOD1), also known as immune-responsive gene 1 (IRG1) [118, 120, 121]. Itaconate inhibits succinate dehydrogenase (SDH), thereby regulating intracellular succinate levels and exerting immunomodulatory effects [122–124]. It also directly alkylates cysteine residues on KEAP1, leading to NRF2 activation and subsequent enhancement of antioxidant and anti-inflammatory responses [125]. In addition, itaconate possess electrophilic properties that regulate the IκBζ–ATF3 inflammatory axis independently of NRF2 [126]. Notably, the anti-inflammatory effects of glucocorticoids have recently been linked to metabolic rewiring of the mitochondrial TCA cycle and increased itaconate production [127]. Accumulated studies uncovered that itaconate or its derivatives, such as cell permeable 4-octyl itaconate (4-OI), can suppress osteoclast differentiation and bone resorption across various pathological contexts, including rheumatoid/inflammatory arthritis [85, 128, 129], steroid-associated osteonecrosis [130], and bone loss [86, 87, 131, 132]. Mechanistically, itaconate exerts its anti-osteoclastogenic effects by inhibiting SDH [85], repressing NLRP3 inflammasome activation [86], and activating NRF2 signalling [87]. IRG1 deletion also enhances NFATc1 expression via upregulation of GRK5 (G protein-coupled receptor kinase 5) [131]. While IRG1 deficiency has minimal impact on bone homeostasis under physiological conditions, it markedly exacerbates osteoclastogenesis and bone resorption following ovariectomy [86] or in inflammatory settings [85]. Recent findings also suggest that itaconate produced during osteoclastogenesis may act in a paracrine manner to stimulate osteoblast differentiation and bone formation [128]. Collectively, these data position itaconate as a key metabolic checkpoint in bone remodelling, with both anti-resorptive and osteoanabolic potential.
3. *Lactate*
  Lactate is generated via glycolysis [133, 134] and is increasingly recognised as a key metabolic regulator for osteoclast formation and function. During osteoclastogenesis, glucose uptake and glycolytic flux are significantly upregulated, with lactate production supporting the energy-intensive bone resorption process [12, 54]. Key glycolytic enzymes, including lactate dehydrogenase A (LDHA) and 6-phosphofructo-2-kinase/fructose-2,6-bisphosphatase 3 (PFKFB3), are significantly upregulated during osteoclastogenesis and mediate lactate production. Inhibition of these enzymes, through either genetic or pharmacological approaches, reduces osteoclast formation and resorptive activity both in vitro and in vivo [88, 135]. Notably, the anti-osteoclastogenic effect of glycolysis inhibition can be reversed by L-lactate supplementation, underscoring lactate’s functional role as a differentiation signal [88]. In bone metastasis models, lactate derived from tumour or immune cells enhances osteoclast recruitment and activation, contributing to metastatic niche formation [136–138]. Elevated LDHA expression in cancer cells is associated with both osteoclast activation and chemoresistance [138]. While high lactate may promote osteoclastogenesis in tumour and inflammatory settings, biofilm-derived lactate does not appear to directly drive osteoclast differentiation, suggesting context-specific effects [139]. Mechanistically, lactate has been shown to promote osteoclastogenic signalling through activation of the NF-κB and MAPK pathways [88]; however, the specific molecular targets of lactate in osteoclasts remain undefined. More recently, lactate has emerged as a key modulator of post-translational modifications (PTMs) in both histone and non-histone proteins [140]. A seminal study by Zhang et al. revealed that lactate can induce histone lactylation, exerting epigenetic regulatory effects [141, 142], which has since garnered significant interest in cancer biology [133, 143, 144]. The regulatory roles of histone and/or protein lactylation in osteoclasts are only beginning to be explored [89] and remain an under-investigated area. Collectively, these findings position lactate as a pro-osteoclastogenic immunometabolite that integrates metabolic, inflammatory, and environmental cues to drive pathological bone loss.
4. *Other immunometabolites*
  Other immunometabolites such as fumarate, tryptophan-derived metabolite (e.g., kynurenine), and short-chain fatty acids (SCFAs) are also important regulators linking metabolism to osteoclast function. Fumarate and its derivative dimethyl fumarate (DMF) exerts anti-inflammatory effects [145] by modifying cysteine residues on target proteins, notably inhibiting NF-κB signalling and activating NRF2-dependent antioxidant responses [146, 147]; its inhibition of osteoclasts is achieved by downregulating NFATc1 and disrupting mitochondrial oxidative metabolism [90, 91]. Kynurenine is a major biologically active metabolite produced from the essential amino acid tryptophan through the enzymatic activity of indoleamine 2,3-dioxygenase 1 (IDO1), and plays a key role in modulating both immune and cancer cell function [148]. It has been shown to promote osteoclast differentiation by activating the aryl hydrocarbon receptor (AhR) pathway, which in turn induces downstream signalling through c-Fos and NFATc1 [92, 93]. Supporting this, in-vivo studies have demonstrated that AhR-deficient mice exhibit impaired osteoclastogenesis and increased bone mass [94, 95]. In addition, SCFAs—including acetate, propionate, and butyrate—are metabolites produced by gut microbiota through fermentation of dietary fibres, and they play complex roles in modulating the host immune system [149]. Acting via the “gut–bone” axis, SCFAs inhibit osteoclast differentiation and exert protective effects on bone homeostasis [96–98]. Together, these metabolites illustrate the expanding landscape of immunometabolites that integrate metabolic and immune signals to influence osteoclast activity.

## Epigenetic Metabolites: Rewiring Osteoclast Gene Regulation

Emerging evidence highlights that cellular metabolism and epigenetic regulation are intimately linked, with key metabolites acting as cofactors or substrates for chromatin-modifying enzymes [150, 151]. During osteoclastogenesis, dynamic metabolic rewiring induces dramatic changes of metabolites which influences the epigenetic landscape, thereby shaping transcriptional programs. Epigenetic metabolites such as alpha-ketoglutarate (αKG), S-adenosylmethionine (SAM), acetyl-coenzyme A (CoA), and nicotinamide adenine dinucleotide (NAD⁺), serve as critical regulators of histone and DNA modifications, integrating environmental and metabolic cues to fine-tune osteoclast gene expression and activity (Table 2; Fig. 2B).

1. *Alpha-KG*
  Intracellular αKG is a central metabolite of the TCA cycle metabolism [152–154]. From an epigenetic perspective, αKG serves as a crucial cofactor for a broad class of αKG-dependent enzymes, including histone lysine demethylase (KDMs) and ten-eleven translocation (TET) enzymes that mediate DNA demethylation [155]. KDMs can lower the level of repressive histone markers, promoting an open chromatin state and enhancing transcriptional activity [155, 156]. Increasing studies suggest that αKG acts as a critical epigenetic regulator during osteoclastogenesis [68, 100]. Stegen et al. recently demonstrated that intracellular αKG production is essential for the activity of the histone demethylase KDM6B, which removes repressive methyl groups from histone H3 lysine 27 (H3K27me3), thereby facilitating the transcription of NFATc1—the master regulator of osteoclast maturation [68]. This finding is consistent with another study showing that αKG supplementation enhances osteoclast formation and bone remodelling in vivo [99]. In contrast, other studies have demonstrated that αKG can negatively regulate osteoclastogenesis [100, 157]. For example, Lee et al. showed that αKG acts as a co-factor to demethylate H3K9 at the promoter of *Slc7a11*, a subunit of the cystine/glutamate antiporter, thereby enhancing Nrf2 binding. This epigenetic regulation suppressed intracellular ROS levels and ultimately inhibited osteoclast differentiation [100]. Of note, the concentrations of αKG used across studies varied, and different levels may differentially affect the activity of αKG-dependent enzymes, potentially altering cellular behaviour through distinct mechanisms. Collectively, these findings suggest that αKG coordinates metabolic and epigenetic signals to regulate osteoclast formation and function, with its stimulatory or inhibitory effects being dose-dependent and context-specific.
2. *SAM*
  SAM is a key metabolite synthesised from the essential amino acid methionine by methionine adenosyltransferase (MAT), through the addition of an adenosine group from ATP [101]. It serves as a universal methyl donor [158], with over 90% of cellular SAM consumed in methylation reactions [101, 102]. From an epigenetic perspective, SAM donates methyl groups for DNA and histone methylation. DNA methylation typically occurs at the 5th carbon of cytosine residues and is catalysed by DNA methyltransferases (DNMTs) [159]. SAM also donates methyl groups for histone methylation via histone methyltransferases (HMTs), targeting specific lysine or arginine residues on histone tails, thereby modulating chromatin structure and transcription [160, 161]. RANKL has been shown to induce metabolic reprogramming toward oxidative metabolism during osteoclast differentiation, which is accompanied by increased production SAM [103, 162, 163]. SAM mediates DNA methylation through DNA methyltransferase 3a (Dnmt3a), leading to the repression of anti-osteoclastic transcription factors such as IRF8 (Interferon Regulatory Factor 8) and enhanced osteoclast differentiation [103]. Dnmt3a deficiency impairs osteoclast formation and results in increased bone mass [103]. More recently, Kang et al. demonstrated that SAM levels are regulated by methionine adenosyltransferase II alpha (MAT2A) during osteoclastogenesis; knockdown of MAT2A led to reduced SAM availability and suppressed the expression of key osteoclastogenic transcription factors, including NFATc1 and c-FOS [164]. Together, these findings underscore the pivotal role of SAM in regulating osteoclastogenesis by integrating epigenetic and metabolic signalling pathways.
3. *Acetyl-CoA*
  Acetyl-CoA is a central hub metabolite that positions at the intersection of catabolic (e.g., glycolysis, fatty acid oxidation) and anabolic metabolism (e.g., lipid synthesis). Acetyl-CoA serves as an essential substrate for histone acetylation—an epigenetic modification that promotes chromatin accessibility and facilitates transcriptional activation, thereby influencing cell fate decisions [165–168]. During osteoclastogenesis, ATP-citrate lyase (ACLY) mediates the conversion of citrate to acetyl-CoA, which contributes to increased histone H3 acetylation (e.g., H3K9, H3K27) and upregulations of osteoclastogenic markers such as NFATc1, c-Fos, and CTSK [104]. In contrast, ACLY inhibition leads to decreased acetyl-CoA availability and reduced H3 acetylation, suppressing osteoclast formation and bone resorption in vitro and in vivo [104]. Recently, Deng et al. revealed that mitochondrial acetyl-CoA production, mediated by the small GTPase Rheb1, is essential for CTSK expression and collagen degradation [105]. Although this study did not investigate epigenetic mechanisms, the observed changes in both CTSK mRNA and protein levels suggest that acetyl-CoA availability might also influence gene regulation through histone modifications. Moreover, accumulating evidence indicates that histone acetylation is a key epigenetic mechanism in osteoclast differentiation [169–172]. As the essential donor of acetyl groups, acetyl-CoA serves as a crucial metabolic regulator, linking cellular metabolic state with epigenetic programming to support osteoclast development and function.
4. *Other epigenetic metabolites*
  Other metabolites, including NAD⁺, flavin adenine dinucleotide (FAD), and 2-hydroxyglutarate (2-HG), function as essential cofactors or inhibitors of epigenetic enzymes and are also increasingly recognised as potential modulators of osteoclasts. NAD⁺ is a crucial cofactor for the sirtuin family of histone deacetylases (e.g., SIRT1), with intracellular NAD⁺ levels directly influencing SIRT activity and thereby affecting histone acetylation [173–175]. NAD⁺ is essential for maintaining osteoclast activity [176], and NAD⁺-dependent sirtuins are emerging as key regulators of osteoclastogenesis. Sirtuins have been found to deacetylate non-histone proteins such as p66Shc [106] and the p65 subunit of NF-κB [107], modulating osteoclast differentiation and function. However, the precise mechanisms by which NAD⁺ regulates the sirtuin family and shapes the epigenetic landscape in osteoclasts remain elusive. Similarly, FAD is a cofactor for the lysine-specific demethylase 1 (LSD1), which demethylates histone (e.g., H3K4, H3K27) [121, 177] and has been implicated in modulating osteoclast-related markers such as TRAP, NFATc1, and CTSK [108–111]. In addition, 2-HG is a derivative of αKG with a structurally similar backbone. 2-HG functions as a competitive inhibitor of αKG-dependent dioxygenases, including KDMs and TETs, thereby inducing epigenetic alterations. 2-HG is well known for its gene-regulatory effects in cancers and is classified as an oncometabolite [112, 113]. Given the established role of αKG in epigenetic regulations during osteoclastogenesis, the epigenetic function of 2-HG in osteoclasts is biologically plausible and warrants further investigation. Together, these metabolites highlight the intricate crosstalk between cellular metabolites and potential epigenetic control of osteoclast differentiation.

## Conclusions and Perspectives

Osteoclastogenesis is a metabolically demanding process that requires precise coordination between cellular metabolism and signalling. Beyond their roles as energy sources and structural components, nutrients and metabolites function as signalling molecules that actively regulate gene expression, epigenetic modifications, organelle function, and membrane dynamics during osteoclastogenesis. Emerging studies have highlighted how metabolic intermediates—such as itaconate, SAM, succinate, and αKG —intersect with canonical pathways and contribute to osteoclast fate decisions. Unravelling the complex and context-dependent roles of metabolites—as messengers, modifiers, and effectors—will be key to developing new therapeutic strategy for skeletal diseases driven by osteoclasts. Additionally, while the importance of nutrients and metabolites for osteoclasts has garnered increasing attention in recent years, currently very little is known about the identities of the transport systems that ferry these small molecules across cellular and organellar membranes in osteoclasts. While some of these molecules are passively transported via simple diffusion, others such as glucose and amino acids often require facilitated transport or energy-dependent active ATP-dependent transport systems to move molecules against their concentration gradients [178]. Recent studies by Ng, Ribet and Guo et al., for example, have shown that that the bioavailability of monosaccharide sugars (e.g. glucose and fructose) is modulated by the lysosomal transport protein Slc37a2 in osteoclasts and that loss of this transporter correlates with disturbances in osteoclast function and bone metabolism [179]. In this regard, defining and characterising the complement of membrane transport proteins that facilitate molecular exchange in osteoclasts remains an important avenue of future research. Similarly, precisely how osteoclasts compartmentalise, mobilise, and utilise nutrients and metabolites in their native tissue microenvironment has remained a long-standing and technically challenging question. Recent advancements in metabolic imaging platforms (e.g. NanoSIMS) [180] coupled with emerging spatial omics technologies [181] have begun to yield important new insights into the biodistribution of nutrients and metabolites at unprecedented subcellular resolution that will help redefine our current understanding of bone metabolism.

## Key References

- Stegen S, Carmeliet G. Metabolic regulation of skeletal cell fate and function. Nat Rev Endocrinol. 2024;20(7):399–413. doi: 10.1038/s41574-024-00969-x IF: 40.0 Q1 B1.
  - ○ This review outlines how skeletal cell metabolism, including in osteoclasts, supports energy demands and cell behaviours, and highlights the emerging role of metabolism as regulators of differentiation, survival, and epigenetic modifications—key themes relevant to osteoclast function and metabolic control.
- Kachler K, Andreev D, Thapa S, Royzman D, Giessl A, Karuppusamy S, et al. Acod1-mediated inhibition of aerobic glycolysis suppresses osteoclast differentiation and attenuates bone erosion in arthritis. Ann Rheum Dis. 2024;83(12):1691-706. doi: 10.1136/ard-2023-224774 IF: 20.6 Q1 B1.
  - ○ This study identifies Acod1 and its metabolite itaconate as negative regulators of osteoclastogenesis. Itaconate inhibits succinate dehydrogenase and HIF-1α-driven glycolysis, limiting osteoclast differentiation and bone erosion in arthritis—highlighting a metabolite-mediated mechanism with therapeutic potential.
- Stegen S, Moermans K, Stockmans I, Thienpont B, Carmeliet G. The serine synthesis pathway drives osteoclast differentiation through epigenetic regulation of NFATc1 expression. Nat Metab. 2024;6(1):141-52. doi: 10.1038/s42255-023-00948-y IF: 20.8 Q1 B1.
  - ○ This study demonstrates that the serine synthesis pathway fuels osteoclastogenesis by generating α-ketoglutarate, which supports histone demethylation at the NFATc1 locus. Inhibiting this pathway impairs osteoclast formation and protects against osteoporosis, revealing a critical metabolic–epigenetic axis in bone resorption.
- Kang H, Guo Q, Dong Y, Peng R, Song K, Wang J, et al. Inhibition of MAT2A suppresses osteoclastogenesis and prevents ovariectomy-induced bone loss. FASEB J. 2022;36(2):e22167. doi: 10.1096/fj.202101205RR IF: 4.2 Q1 B2.
  - ○ This study identifies MAT2A as a key regulator of osteoclastogenesis via SAM production. Inhibiting MAT2A reduces SAM levels, suppresses NF-κB signalling and ROS production, and impairs osteoclast differentiation and bone resorption—highlighting MAT2A as a potential therapeutic target in osteoporosis.
- Guo Q, Kang H, Wang J, Dong Y, Peng R, Zhao H, et al. Inhibition of ACLY Leads to Suppression of Osteoclast Differentiation and Function Via Regulation of Histone Acetylation. J Bone Miner Res. 2021;36(10):2065-80. doi:10.1002/jbmr.4399 IF: 5.9 Q1 B1.
  - ○ This study demonstrates that ACLY promotes osteoclastogenesis by generating acetyl-CoA for histone H3 acetylation. ACLY inhibition impairs osteoclast differentiation and bone resorption, suggesting ACLY and acetyl-CoA levels are metabolic-epigenetic target for bone loss treatment.
- Deng D, Liu X, Huang W, Yuan S, Liu G, Ai S, et al. Osteoclasts control endochondral ossification via regulating acetyl-CoA availability. Bone Res. 2024;12(1):49. doi: 10.1038/s41413-024-00360-6 IF: 15.0 Q1 B1.
  - ○ This study identifies mitochondrial acetyl-CoA as a key regulator of cathepsin K (CTSK) expression in osteoclasts, independent of mTORC1. Rheb1 controls this metabolic pathway, linking osteoclast mitochondrial respiration to fracture healing, with implications for targeting metabolic dysfunction in bone disorders.

## Data Availability

No datasets were generated or analysed during the current study.
